# Supplementary material for: Unveiling Neuronal Microstructure in the Human Brain In Vivo With Time‐Dependent Radial Diffusivity in a Head‐Only High‐Performance Gradient 3T MRI System
Source: Hum Brain Mapp. 2026 Nov 12;47(16):e70664. doi: 10.1002/hbm.70664 (PMC13756840; doi:10.1002/hbm.70664)
Supplement: Supplementary file 1 [file HBM-47-e70664-s001.docx]

***Supporting Figure S1****. Mean ΔRD/Δf of each white matter parcel in Subject #1, Subject #2, and Subject #3. ΔRD/Δf measured from the five time-dependent DTI protocols is shown for each white matter parcel.*

***Supporting Table S1****.* *ΔRD/Δf measurements in the white matter parcels associated with the corticospinal tract, forceps minors and forceps majors. Parcels from the left (L) and right (R) hemispheres are shown separately. Results are presented for each healthy subject (Subjects #1, #2, #3), along with the mean ΔRD/Δf across all subjects. The unit of ΔRD/Δf is μm^2^.*

|  |  | $\frac{\Delta RD(100 Hz b800)}{100 Hz}$ | $\frac{\Delta RD(85 Hz b1000)}{85 Hz}$ | $\frac{\Delta RD(85 Hz b500)}{85 Hz}$ | $\frac{\Delta RD(54 Hz b1000)}{54 Hz}$ | $\frac{\Delta RD(28 Hz 500)}{28 Hz}$ |
| --- | --- | --- | --- | --- | --- | --- |
| Corticospinal tract | Atlas-defined Corticospinal tract ROI  (CST.L / CST.R) | L: 1.68, 2.48, 2.08  R: 2.04, 2.32, 1.92 | L: 1.82, 2.44, 2.24  R: 1.88, 2.27, 1.98 | L: 1.16, 1.72, 1.34  R: 1.29, 1.73, 1.39 | L: 1.17, 1.59, 1.35  R: 0.93, 1.48, 1.33 | L: 0.86, 2.04, 0.68  R: 1.71, 2.57, 1.07 |
|  |  | L: 2.04 / R: 2.13 | L: 2.15 / R: 2.05 | L: 1.51 / R: 1.52 | L: 1.46 / R: 1.33 | L: 1.39 / R: 1.82 |
|  | Superior corona radiata (SCR.L / SCR.R) | L: 1.92, 2.13, 2.20  R: 1.88, 2.05, 2.11 | L: 1.93, 2.11, 2.17  R: 1.97, 2.13, 2.17 | L: 1.62, 1.55, 1.68  R: 1.71, 1.56, 1.62 | L: 1.41, 1.37, 1.69  R: 1.33, 1.41, 1.50 | L: 1.71, 2.14, 2.21  R: 2.11, 2.36, 2.00 |
|  |  | L: 2.03 / R: 1.97 | L: 2.05 / R: 2.04 | L: 1.61 / R: 1.60 | L: 1.46 / R: 1.39 | L: 2.07 / R: 2.21 |
|  | Posterior corona radiata (PCR.L / PCR.R) | L: 1.31, 1.56, 1.62  R: 1.26, 1.57, 1.49 | L: 1.41, 1.72, 1.64  R: 1.40, 1.57, 1.66 | L: 1.11, 1.27, 1.29  R: 1.18, 1.15, 1.22 | L: 1.06, 1.13, 1.24  R: 1.02, 1.09, 1.13 | L: 1.68, 2.29, 1.82  R: 1.54, 1.93, 1.82 |
|  |  | L: 1.50 / R: 1.43 | L: 1.58 / R: 1.54 | L: 1.20 / R: 1.20 | L: 1.15 / R: 1.07 | L: 1.93 / R: 1.79 |
|  | Posterior limb of the internal capsule  (PLIC.L / PLIC.R) | L: 1.96, 1.99, 2.08  R: 2.02, 2.03, 1.95 | L: 1.99, 2.15, 1.94  R: 2.08, 2.06, 1.87 | L: 1.75, 1.58, 1.58  R: 1.75, 1.53, 1.36 | L: 1.43, 1.35, 1.50  R: 1.39, 1.31, 1.37 | L: 1.50, 1.86, 1.96  R: 1.57, 2.21, 1.82 |
|  |  | L: 2.07 / R: 1.96 | L: 2.05 / R: 1.99 | L: 1.68 / R: 1.54 | L: 1.43 / R: 1.35 | L: 1.82 / R: 1.93 |
|  | Cerebral Peduncle  (CP.L / CP.R) | L: 1.33, 1.72, 1.41  R: 1.04, 1.34, 1.00 | L: 1.62, 1.44, 1.34  R: 1.13, 1.32, 1.55 | L: 1.32, 1.08, 0.89  R: 0.93, 1.05, 0.85 | L: 1.04, 1.11, 1.30  R: 1.13, 1.30, 1.13 | L: 1.11, 1.54, 1.32  R: 1.54, 1.39, 1.86 |
|  |  | L: 1.70 / R: 1.42 | L: 1.58 / R: 1.60 | L: 1.24 / R: 1.25 | L: 1.19 / R: 1.28 | L: 1.46 / R: 2.18 |
|  | Pontine crossing tract (PCT) | 1.61, 1.29, 1.56 | 1.52, 1.45, 1.75 | 1.67, 1.67, 1.72 | 1.57, 1.26, 1.54 | 4.18, 3.46, 3.50 |
|  |  | 1.48 | 1.59 | 1.67 | 1.44 | 3.75 |
| Forceps minors | Genu of corpus callosum (gCC) | 0.74, 0.65, 0.86 | 0.95, 0.91, 0.99 | 0.91, 0.53, 0.74 | 0.80, 1.02, 0.94 | 1.04, 0.32, 1.07 |
|  |  | 0.84 | 1.01 | 0.82 | 0.89 | 1.04 |
|  | Anterior corona radiata (ACR.L / ACR.R) | L: 1.10, 1.14, 1.15  R: 1.10, 1.19, 1.29 | L: 1.24, 1.24, 1.28  R: 1.26, 1.22, 1.25 | L: 0.96, 0.72, 0.82  R: 1.04, 0.76, 0.92 | L: 0.87, 0.74, 0.91  R: 0.89, 0.74, 0.89 | L: 1.25, 1.29, 0.82  R: 1.79, 1.54, 1.18 |
|  |  | L: 1.15 / R: 1.21 | L: 1.28 / R: 1.26 | L: 0.86 / R: 0.93 | L: 0.89 / R: 0.87 | L: 1.11 / R: 1.54 |
| Forceps majors | Splenium of corpus callosum (sCC) | 0.90, 1.10, 1.44 | 1.05, 1.05, 1.37 | 1.04, 0.92, 1.21 | 0.80, 0.85, 1.07 | 0.82, 0.64, 0.93 |
|  |  | 1.22 | 1.21 | 1.11 | 0.93 | 1.04 |
|  | Tapetum (TAP.L / TAP.R) | L: -0.20, 0.96, -0.62  R: 0.40, -0.03, 0.66 | L: 0.20, 2.27, 0.17  R: 0.89, 0.61, 0.95 | L: 0.34, -0.18, -0.05  R: 0.71, -0.55, 0.40 | L: -0.04, 1.44, -0.02  R: 0.56, 0.30, 0.85 | L: -1.43, -1.50, -2.79  R: -0.36, -5.64, -1.07 |
|  |  | L: 0.47 / R: 0.82 | L: 0.91 / R: 1.09 | L: 0.56 / R: 0.80 | L: 0.72 / R: 0.83 | L: -0.32 / R: 0.11 |
|  | Sagittal stratum  (SS.L / SS.R) | L: 1.10, 1.20, 1.05  R: 0.68, 1.09, 0.99 | L: 1.25, 1.25, 1.18  R: 0.99, 1.29, 1.11 | L: 0.99, 0.87, 0.93  R: 0.64, 0.79, 0.82 | L: 0.81, 0.74, 1.00  R: 0.80, 0.72, 0.89 | L: 1.36, 1.36, 1.57  R: 0.57, 1.64, 0.86 |
|  |  | L: 1.15 / R: 0.99 | L: 1.27 / R: 1.12 | L: 0.93 / R: 0.85 | L: 0.85 / R: 0.81 | L: 1.39 / R: 1.29 |
|  | Posterior thalamic radiation (PTR.L / PTR.R) | L: 1.13, 1.11, 1.24  R: 1.09, 1.04, 1.22 | L: 1.15, 1.22, 1.28  R: 1.19, 1.15, 1.37 | L: 1.05, 0.86, 0.94  R: 1.06, 0.75, 1.11 | L: 0.96, 0.61, 0.91  R: 0.93, 0.72, 0.98 | L: 1.29, 1.50, 1.82  R: 1.07, 1.21, 1.36 |
|  |  | L: 1.21 / R: 1.19 | L: 1.26 / R: 1.27 | L: 0.95 / R: 1.01 | L: 0.87 / R: 0.93 | L: 1.61 / R: 1.32 |
| All-subject mean calculated by averaging DRD of all voxels in the white matter parcel from all three healthy subjects. L: left; R: right. | | | | | | |
